# Transcriptome reveals the roles and potential mechanisms of CeRNA in the regulation of salivary gland development in the tick *Rhipicephalus haemaphysaloides*

**DOI:** 10.3389/fcimb.2025.1573239

**Published:** 2025-04-30

**Authors:** Shanming Hu, Songqin Chen, Haotian Zhu, Yanan Wang, Yongzhi Zhou, Jie Cao, Houshuang Zhang, Jinlin Zhou

**Affiliations:** ^1^ Key Laboratory of Animal Parasitology of Ministry of Agriculture, Shanghai Veterinary Research Institute, Chinese Academy of Agricultural Sciences, Shanghai, China; ^2^ College of Animal Science and Technology, Anhui Agricultural University, Hefei, China

**Keywords:** salivary gland, ceRNA, tick, miRNA, lncRNA

## Abstract

**Introduction:**

The salivary glands of female ticks rapidly degenerate after feeding. The mechanism involves programmed cell death mediated by an ecdysteroid receptor. A competing endogenous RNA (ceRNA) network has been established using miRNA and the competitive binding of three types of RNA (lncRNA, circRNA, and mRNA), that were demonstrated to be involved in the regulation of biological processes. However, the comprehensive expression profile and competing endogenous RNA (ceRNA) regulatory network between mRNAs and ncRNAs involved in salivary gland development remain unclear.

**Methods:**

In the current study, we employed whole-transcriptome sequencing (RNA sequencing) at various stages of feeding to identify differentially expressed lncRNAs, circRNAs, miRNAs, and mRNAs. The ceRNA networks combining lncRNAs, circRNAs, miRNAs, and mRNAs were predicted and constructed based on the miRanda and TargetScan databases. Gene ontology (GO) and Kyoto Encyclopedia of Genes and Genomes (KEGG) pathway analyses were performed for target mRNAs with significantly different expression levels.

**Results:**

We identified several pathways related to organ growth and development: Insulin secretion, the Hippo signaling pathway, the Pl3K-Akt signaling pathway, the FoxO signaling pathway, and the Ferroptosis pathway in the lncRNA-miRNA-mRNA network, and Steroid biosynthesis, Cholesterol metabolism, the FoxO signaling pathway, and the Ferroptosis pathway in the circRNA-miRNA-mRNA network, each of which involved insulin and ecdysteroid regulation.

**Discussion:**

Our findings have advanced our understanding of the underlying mechanisms of salivary gland development and degeneration.

## 1 Introduction

Ticks are obligatory blood-feeding arthropods that act as vectors for many important pathogens (Jongejan and Uilenberg, 2004). Ticks may remain attached to their hosts and feed for several days or even weeks (Sonenshine, 1991). The salivary glands of ticks mediate diverse functions involved in survival and reproduction (Simo et al., 2017), and tick-borne pathogens (TBPs) are transmitted to the host via the saliva during feeding (Kazimírová and Stibrániová, 2013).

The feeding period of female ticks is divided into three phases: (i) a preparatory phase, during which the feeding lesion is established, (ii) a slow phase, during which tick weight increases by up to 10-fold over the unfed weight, and (iii) a rapid phase, during which tick weight demonstrates a further 10-fold increase (Mao and Kaufman, 1999a). At the transition between the slow and rapid phases of engorgement, there is a marked change in the salivary glands. During the slow phase feeding, the salivary glands of Ixodid females develop rapidly, so that majority of the salivary gland acinar cells undergo marked hypertrophy, resulting in an overall increase in the mass of the salivary glands (Simo et al., 2017). Salivary gland degeneration begins at the rapid phase of feeding, possibly due to apoptosis and autophagy-related to the increase in ecdysone in the hemolymph (Mao and Kaufman, 1999a; Francischetti et al., 2009; Abdelwahid et al., 2011). During the degeneration, the granular acini undergo DNA fragmentation, and caspase enzyme activity increases (L’Amoreaux et al., 2003; Freitas et al., 2007). A comprehensive analysis of the changes in protein levels showed that those associated with apoptosis and autophagy were altered in expression, with enzymes linked to the degradation of DNA and proteins being consistently upregulated (Wang et al., 2019b). A recent study identified three caspases (RhCaspases7, RhCaspases8, and RhCaspases9) involved in the degeneration of the salivary glands of In *R. haemaphysaloides* (Wang et al., 2020).

Further research has demonstrated that silencing the ecdysteroid receptors (ECRs) inhibits salivary gland degeneration by affecting caspase-dependent apoptosis (Lu et al., 2021). The ECR is activated by 20-hydroxyecdysone (20E), a commonly occurring steroid hormone, and coordinates multiple developmental events, including molting, diapause, spermatogenesis, and salivary gland degeneration. In arthropods, ecdysteroid hormones, including 20E, are the primary steroid hormones essential for successful molting and metamorphosis. Ecdysteroid hormones influence arthropod development and reproduction by binding a heterodimeric complex of nuclear receptors, including the ecdysone receptor (EcR) and ultraspiracle (USP) (Yan et al., 2016). While the molecular mechanism underlying the ecdysteroid receptor is relatively well-known in insects, less is known about the molecular mechanisms of ecdysteroid action in ticks. Recent studies, however, have shed light on this process. For instance, deep sequencing of small RNA libraries for ticks has revealed that the let-7 microRNA can regulate the expression of the ECR to influence molting (Wu et al., 2019; Liu et al., 2020a). This discovery highlights the importance of small non-coding RNAs (ncRNAs) in regulating gene expression and developmental processes.

Building on this understanding, miRNAs are small ncRNA molecules that can silence RNA and regulate post-transcriptional gene expression (Bartel, 2004). Furthermore, a competing endogenous RNA (ceRNA) network can be established through micro (mi)RNAs and the competitive binding of three types of RNA (long non-coding (lnc)RNAs, circular (circ)RNAs and mRNA) that provides a method of identifying key RNAs that affect biological processes (Jiang et al., 2015; Taborda et al., 2017; Li et al., 2018). LncRNAs are RNA molecules with a length of more than 200 nucleotides; these do not encode proteins but are involved in transcriptional regulation by binding to miRNA (Guttman et al., 2009; Wang et al., 2019a; Yi et al., 2019). CircRNA is a class of conserved and structurally stable non-coding RNAs that can act as a miRNA sponge to regulate mRNA expression, cell migration, and invasion (Memczak et al., 2013). In recent years, analysis of the full transcriptome has gradually become the dominant method for studying the mechanisms of arthropod development. Transcriptome analysis has been employed to investigate the roles of ncRNAs in regulating *Drosophila* accessory gland development, male fertility, and the reproductive capacity of the Asian tiger mosquito (Maeda et al., 2018; Belavilas-Trovas et al., 2022). Specifically, lncRNA-yar has been identified as a crucial regulator of *Drosophila* sleep homeostasis through its interaction with miRNAs (Soshnev et al., 2011). Additionally, a circRNA, *ame_circ_2015*, has been found to promote egg laying in *Apis. mellifera* by negatively regulating miR-14, a miRNA known to modulate the ecdysteroid pathway and thereby reduce egg production (Chen and Fu, 2021). However, its application in understanding how lncRNAs regulate tick growth and development remains unexplored.

In this study, a transcriptome analysis was performed to profile the mechanism of CeRNAs (lncRNA-miRNA-mRNA or circRNA-miRNA-mRNA) underlying salivary gland degeneration in ticks. This study provides insight into the physiological roles of CeRNAs in gene regulatory networks as well as their biological functions in *R. haemaphysaloides*.

## 2 Materials and methods

### 2.1 Tick feeding and tissue collection

Adult *R. haemaphysaloides* were collected in May 2001 from a water baffle in Wuhan, Hubei Province, China. The ticks were fed on New Zealand white rabbits (SLAC, Shanghai Institutes for Biological Science, CAS) and maintained in artificial climate incubators at the Shanghai Veterinary Research Institute (Zhou et al., 2006). Tick salivary glands at different feeding times were dissected, washed with phosphate-buffered saline (PBS, PH 7.4, with 0.14 M NaCl and 0.0027 M KCl, 0.01 M phosphate buffer; (Gibco, Life Technologies, Carlsbad, CA, USA), and placed in PBS or TRIzol (Invitrogen, Carlsbad, CA, USA) reagent at −80°C. Samples of the salivary glands were taken at different feeding stages (not feeding, feeding for five days, and engorged for three days) and collected from 15 ticks per group. Total RNA was extracted using a TRIzol reagent kit (Invitrogen, Carlsbad, CA, USA) according to the manufacturer’s protocol.

### 2.2 MiRNA sequence analysis

The RNA was sequenced using an Illumina HiSeq Xten platform by Gene Denovo Biotechnology Co. (Guangzhou, China). All of the clean tags were aligned with small RNAs in the GeneBank (Release 209.0) and Rfam (Release 11.0) databases to identify and remove rRNA, scRNA, snRNA, snoRNA, and tRNA. Differential expression (DE) miRNA analysis between groups was performed by edgeR software. We identified miRNAs with a fold change (FC) ≥ 2 and *P* value < 0.05 as differentially expressed miRNAs (DEmRNAs). Two software programs, Miranda (Version 3.3a) and TargetScan (Version 7.0), were used to predict RNA targets. The Gene Ontology (GO) (http://www.geneontology.org/) and Kyoto Encyclopedia of Genes and Genomes (KEGG) (https://www.kegg.jp) databases were used for the functional annotation of target mRNAs (Kanehisa et al., 2008). GO terms or pathways with *P* < 0.05 were defined as significantly enriched GO terms or pathways. These processes are illustrated in the workflow diagram (**Supplementary Figure S1**).

### 2.3 mRNA, lncRNA, and circRNA sequence analysis

Total RNA was obtained from salivary gland tissue. RNA quality was assessed on an Agilent 2100 Bioanalyzer (Agilent Technologies, Palo Alto, CA, USA) and checked using RNase-free agaro se gel electrophoresis. To obtain high-quality clean reads, the reads were further filtered by fastp (version 0.18.0). The clean data were obtained by removing low quality reads containing more than 10% poly(N) or more than 50% low-quality (Q-value ≤ 20) bases and adapter reads from the raw data. The short reads alignment tool Bowtie2 (version 2.2.8) was used for mapping reads to the rRNA databases. The coding ability of the new transcripts was predicted by two software programs, CPC and CNCI. Differential expression analysis of mRNAs and ncRNAs was determined using DESeq2. The mRNAs/ncRNAs with a false discovery rate (FDR) < 0.05/absolute FC ≥ 1.5 and *P* value < 0.05/FC ≥ 2 were considered DEmRNAs/DEncRNAs, respectively. DEmRNAs were then subjected to enrichment analysis of GO functions and KEGG pathways. The GO and KEGG analyses were performed on DEncRNAs after the prediction of target genes. These processes are illustrated in the workflow diagram (**Supplementary Figure S2**).

### 2.4 ceRNA regulation analysis

Spearman rank correlation was used to evaluate the correlations in expression levels between mRNA-miRNA, circRNA-miRNA, and lncRNA-miRNA. Pairs with SRCC < 0.7 were chosen as co-expressed negative pairs. DEmRNA, DEcircRNA, and DElncRNA were DEmiRNA target genes. All the co-expression competitive triplets identified above were assembled into a lncRNA-miRNA-mRNA and circRNA-miRNA-mRNA network and visualized using Cytoscape software (v3.6.0) (http://www.cytoscape.org/).

### 2.5 CeRNA connectivity analysis in salivary gland development pathways

A pathway connectivity network visualization analysis was performed using Cytoscape software for all key RNAs enriched in resistance-related pathways. The RNA molecules and hub genes strongly related to salivary gland development in *R. haemaphysaloides* were identified.

### 2.6 Validation of miRNA and lncRNA expression by quantitative reverse transcription polymerase chain reaction

Six lncRNAs and six miRNAs identified as differentially expressed were selected, and the RNA used for qRT–PCR was the same as that used for sequencing. lncRNA primers were designed with Oligo7 based on the lncRNA sequences (**Additional File** **Supplementary Table S5**) to ensure specificity and avoid overlap with the target genes. The lncRNA sequences were amplified using the PrimeScript™ RT Reagent Kit with gDNA Eraser (Takara Bio). The miRNA first-strand cDNA was synthesized using the Mir−X miRNA First-Strand Synthesis Kit (Takara Bio). The RT primers for miRNA are listed in **Supplementary Table S6**.

The elongation factor-1(ELF1A, Genbank accession no. AB836665) gene was chosen as an internal control for lncRNA. U6 was used for miRNA (Ma et al., 2018; Zheng et al., 2019). All samples were analyzed in triplicate. The relative expression levels of genes were calculated by the 2^-δδCt^ method, and the mean and standard deviation of three biological replicates are shown as the results.

### 2.7 Statistical analysis

Student’s t test was used to compare means for the RNA-sequencing data. The differences with fold change (FC) > 2 and P < 0.05 were considered to be statistically significant in the RNA-sequencing analysis. FDR was calculated to correct the *P* values in the RNA-sequencing analysis. The expression level of each mRNA, miRNA, lncRNA, and circRNA was represented as a fold change using the 2-δδCt method for real-time qPCR analysis. P < 0.05 was considered statistically significant.

## 3 Results

### 3.1 Profile characteristics of sRNAs and nucleotide bias of potential miRNAs from *R. haemaphysaloides*

After deep sequencing using the Hiseq 4000 platform (Illumina), Filtering and adapter trimming were performed on the data (**Supplementary Table S1**). Approximately 109,125,795 reads were obtained, and nine sRNA libraries were constructed for *R. haemaphysaloides* (**Supplementary Table S2**). The nine libraries were annotated with tags for rRNA, snoRNA, snRNA, tRNA, known miRNA, and novel miRNA derived from the GenBank and Rfam databases. The analysis resulted in 12,777,574 reads for the unfed group, 11,860,938 reads for the 5 days (F5d) group, and 11,733,753 reads for the engorged 3 days (E3d) group. After filtering, approximately 5,702,508 reads for the unfed group, 4,936,045 reads for the F5d group, and 4,531,089 reads for the E3d group were approved for known miRNA analysis. 543,484 reads for the unfed group, 158,001 reads for the F5d group, and 148,195 reads for the E3d group were approved for novel miRNA analysis.

### 3.2 Identification of differentially expressed miRNAs

**Figure 1:**
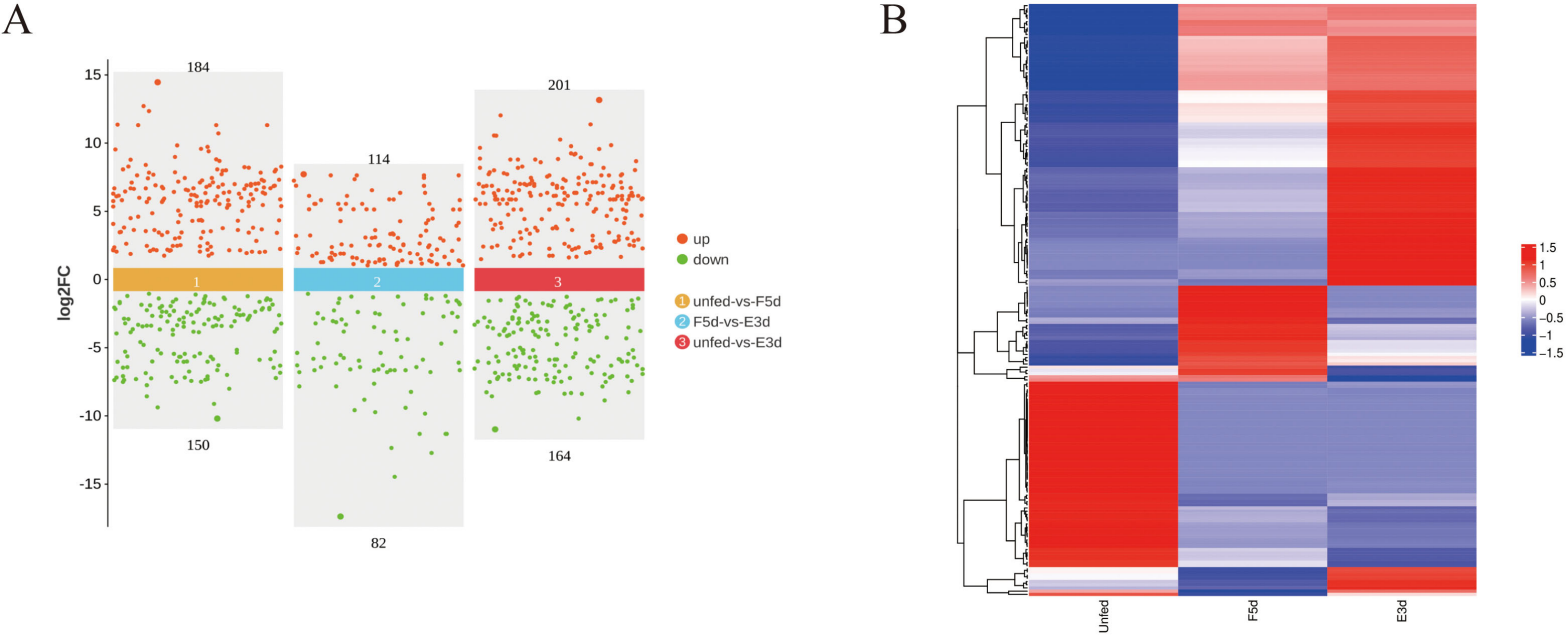
Differentially expressed miRNAs analysis between unfed, F5d, and E3d groups **(A)** Scatter diagram of significantly differentially expressed miRNAs between the unfed, F5d, and E3d groups. Red and green dots represent upregulated and downregulated miRNAs, respectively. **(B)** Cluster heatmap of differential expression miRNAs between the unfed, F5d, and E3d groups.

A total of 499 differentially expressed miRNAs were identified in the salivary gland tissue. A volcano plot was generated to illustrate the expression levels of miRNAs in the unfed, F5d, and E3d groups (**Figure 1A**), and a heatmap was generated to compare the expression patterns of the miRNAs. The results indicated that most miRNAs were increased during salivary gland development at the various feeding stages (**Figure 1B**). Of these differentially expressed miRNAs, 184 were upregulated, and 150 were downregulated in the unfed group compared to the F5d group; 114 were upregulated and 82 were downregulated in the unfed group compared to the E3d group, and 201 were upregulated and 164 were downregulated in the F5d group compared to the E3d group.

**Figure 2:**
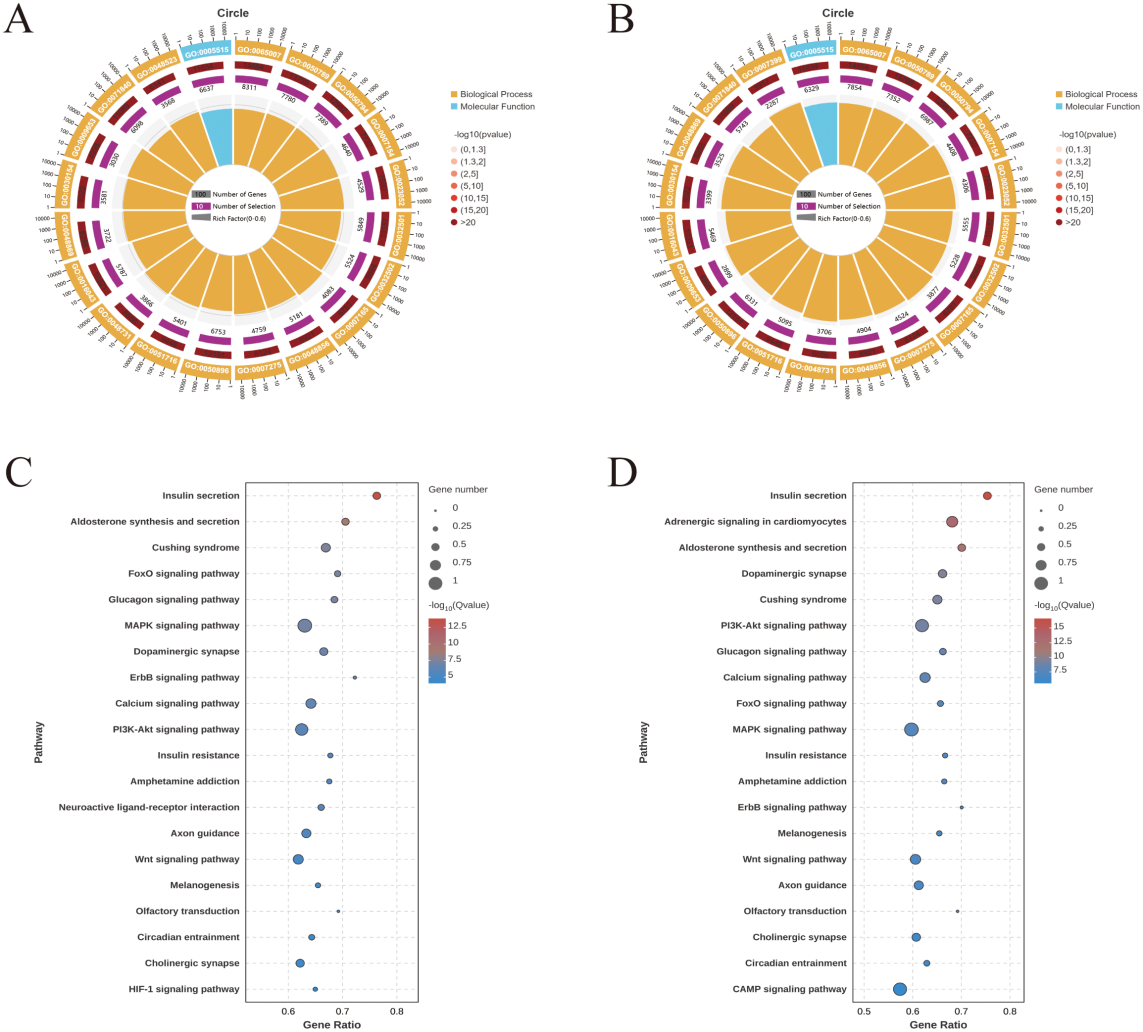
GO and KEGG enrichment analyses for predicted target genes of the differentially expressed miRNAs expressed during feeding stages of *R. haemaphysaloides* (the top 20). **(A)** GO enrichment of the unfed group vs. the F5d group. **(B)** GO enrichment of the F5d group vs. the E3d group. GO functional annotations in the three main categories: molecular function, cellular component, and biological process. **(C)** KEGG enrichment of the unfed group vs. the F5d group. **(D)** KEGG enrichment of the F5d group vs. the E3d group.

For the unfed vs. F5d group and F5d vs. E3d group comparisons, the Gene Ontology functional annotations of target mRNAs of miRNAs were largely involved with biological regulation (GO:0065007), regulation of cellular process (GO:0050794), developmental process (GO:0032502), cellular response to stimulus (GO:0051716) and protein binding (GO:0005515) (**Figures 2A, B**).

Target genes were mapped to the KEGG database to identify signaling pathways during different feeding stages. The top 20 highly enriched pathways in the unfed, unfed vs. F5d, and F5d vs. E3d group comparisons are shown in **Figures 2C, D**. Notably, several pathways related to organ growth and development were identified: Insulin secretion, the Calcium signaling pathway, the Glucagon signaling pathway, the FoxO signaling pathway, the PI3K-Akt signaling pathway, the MAPK signaling pathway and the Wnt signaling pathway.

### 3.3 Identification of differentially expressed lncRNAs and circRNAs

**Figure 3:**
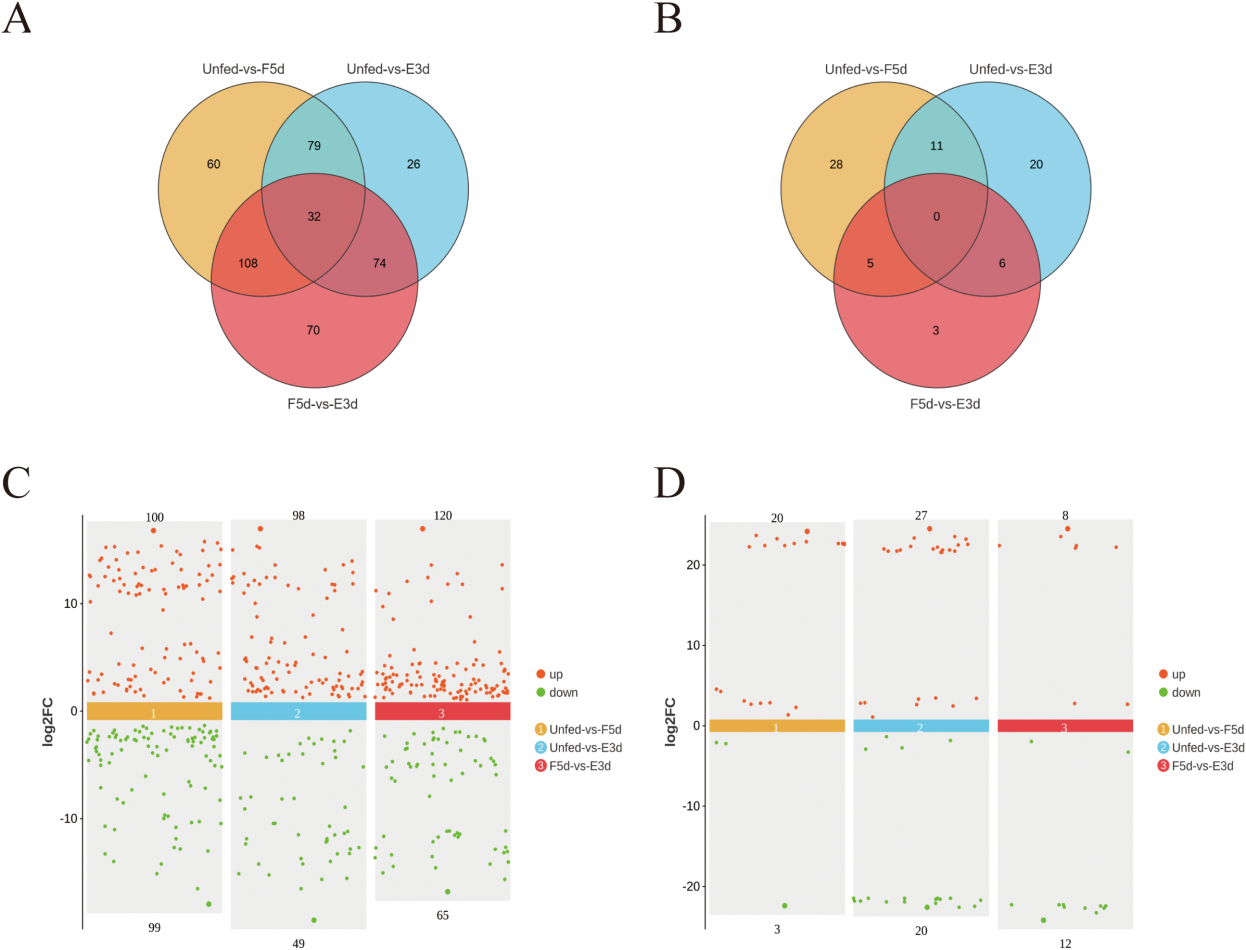
Differentially expressed lncRNA and circRNAs analysis between the unfed, F5d, and E3d groups **(A)** Venn diagram showing significantly differentially expressed lncRNA transcripts between the unfed, F5d, and E3d groups. **(C)** scatter diagram of significantly differentially expressed lncRNAs between the unfed, F5d, and E3d groups. **(B)** Venn diagram showing significantly differentially expressed circRNAs transcripts between the unfed, F5d, and E3d groups. **(D)** Scatter diagram of significantly differentially expressed circRNAs between the unfed, F5d, and E3d groups. Red and green dots represent upregulated and downregulated lncRNAs and circRNAs, respectively.

After the sequencing data were subjected to low-quality filtering (**Supplementary Table S3**) and subsequent alignment (**Supplementary Table S4**), a total of 449 known and novel lncRNAs without preexisting annotation information were identified in the unfed, F5d, and E3d groups. A Venn diagram was constructed based on the expression levels of these lncRNAs to determine the intersection of the expression levels within each group (any lncRNAs with a count value greater than 0 in two or more samples were considered to be expressed in that group) (**Figure 3A**). The lncRNAs for which the false discovery rate (FDR) was less than 0.05 and the absolute value of the fold change was ≥ 2 were considered differentially expressed. Of these differentially expressed lncRNAs, 100 were upregulated and 99 were downregulated in the unfed group compared to the F5d group; 98 were upregulated and 49 were downregulated in the unfed group compared to the E3d group, and 120 were upregulated and 65 were downregulated in the F5d group compared to the E3d group (**Figure 3C**).

A total of 73 differentially expressed circRNAs were obtained under the screening criteria. These were the sum of differentially expressed circRNAs in the three groups (the unfed, F5d, and E3d groups) after comparisons between group pairs (**Figure 3B**). Differentially expressed circRNAs in the samples are shown in a Volcano plot (**Figure 3D**). Twenty were upregulated and three were downregulated in the unfed group compared to F5d group; 27 were upregulated and 20 were downregulated in the unfed group compared to the E3d group, and eight were upregulated and 12 were downregulated in the F5d group compared to the E3d group.

### 3.4 Response of the CeRNA regulatory network to salivary gland development

**Figure 4:**
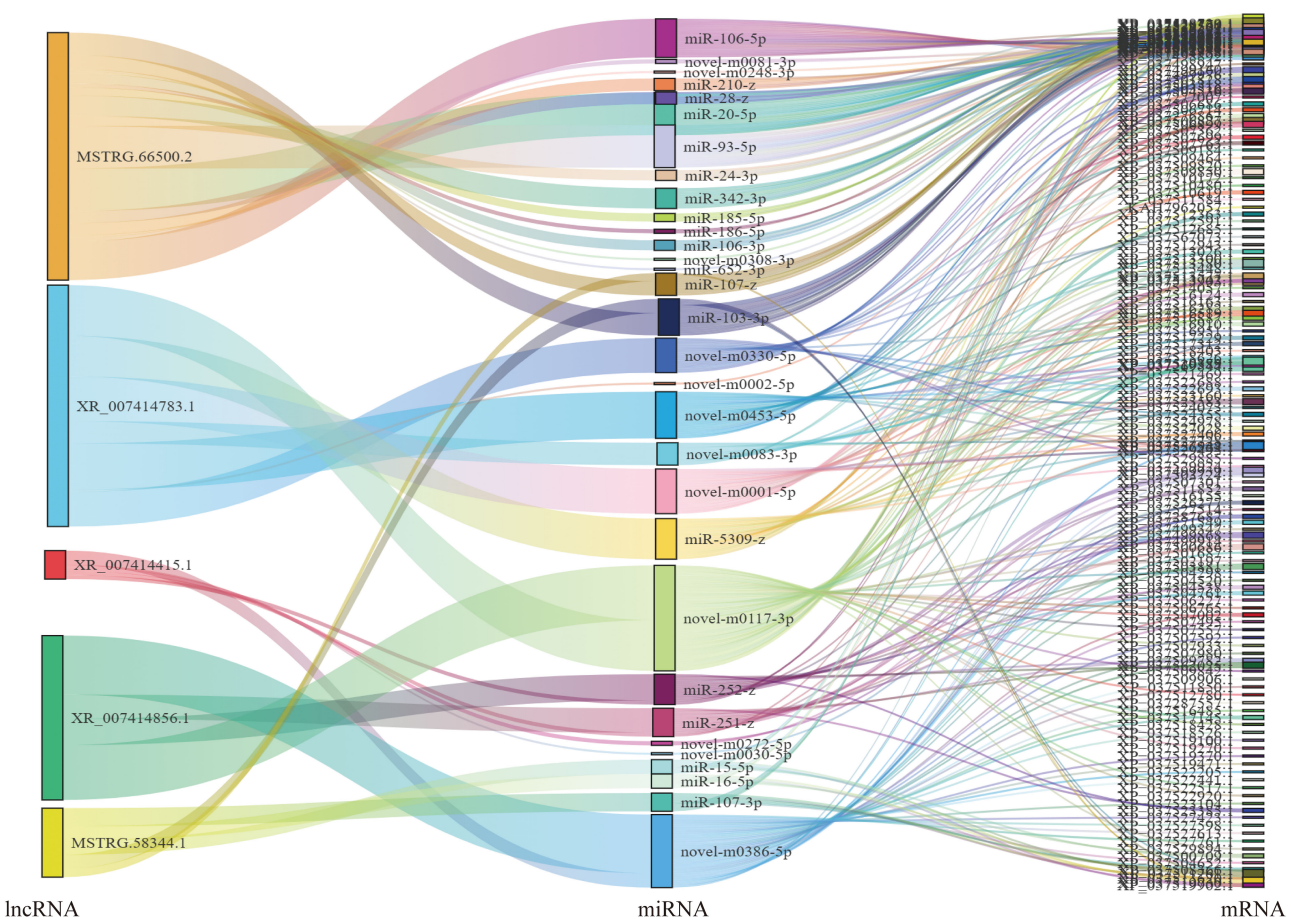
Association analysis of lncRNA–miRNA–mRNA. A Sankey diagram of the lncRNA connectivity (top 5);.

Based on the ceRNA model, Cytoscape software was used to visualize the results of the mutual targeting regulatory network of all the significantly differentially expressed circRNAs, lncRNAs, miRNAs, and mRNAs. The network comprised 59 lncRNAs targeting 104 miRNAs that in turn bound to 368 mRNAs, and seven circRNA targeting 18 miRNAs that in turn bound to 58 mRNAs. A Sankey diagram showing the top five differentially expressed lncRNA and seven circRNA connectivity was created according to the function prediction and targets of key differentially expressed mRNAs (**Figure 4**).

**Figure 5:**
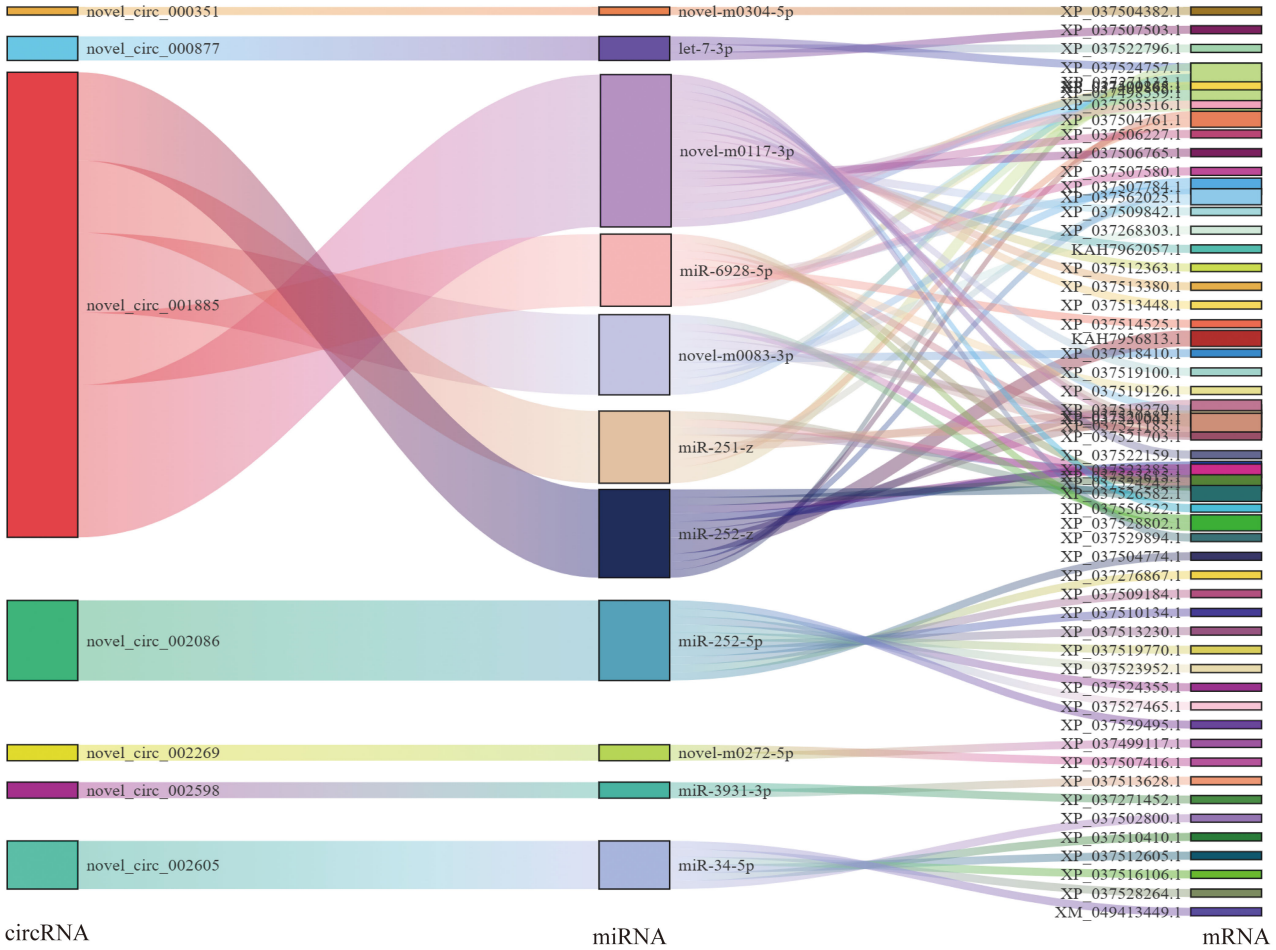
Association analysis of circRNA–miRNA–mRNA interactions. A Sankey diagram of the circRNA connectivity.

For the lncRNA–miRNA–mRNA, MSTRG.66500.2 bound the largest number of miRNAs. There were 16, 7, 5, 5, and 4 regulatory connections to the top five nodes (MSTRG.66500.2, XR_007414783.1, XR_007414415.1, MSTRG.58344.1 andXR_007414856.1) Among the circRNA–miRNA–mRNA, novel_circ_001885 bound five miRNAs, other circRNAs bound one miRNA (novel_circ_000351, a novel_circ_000877, a novel_circ_002086, a novel_circ_002269, novel_circ_002598, and novel_circ_002605) (**Figure 5**).

**Figure 6:**
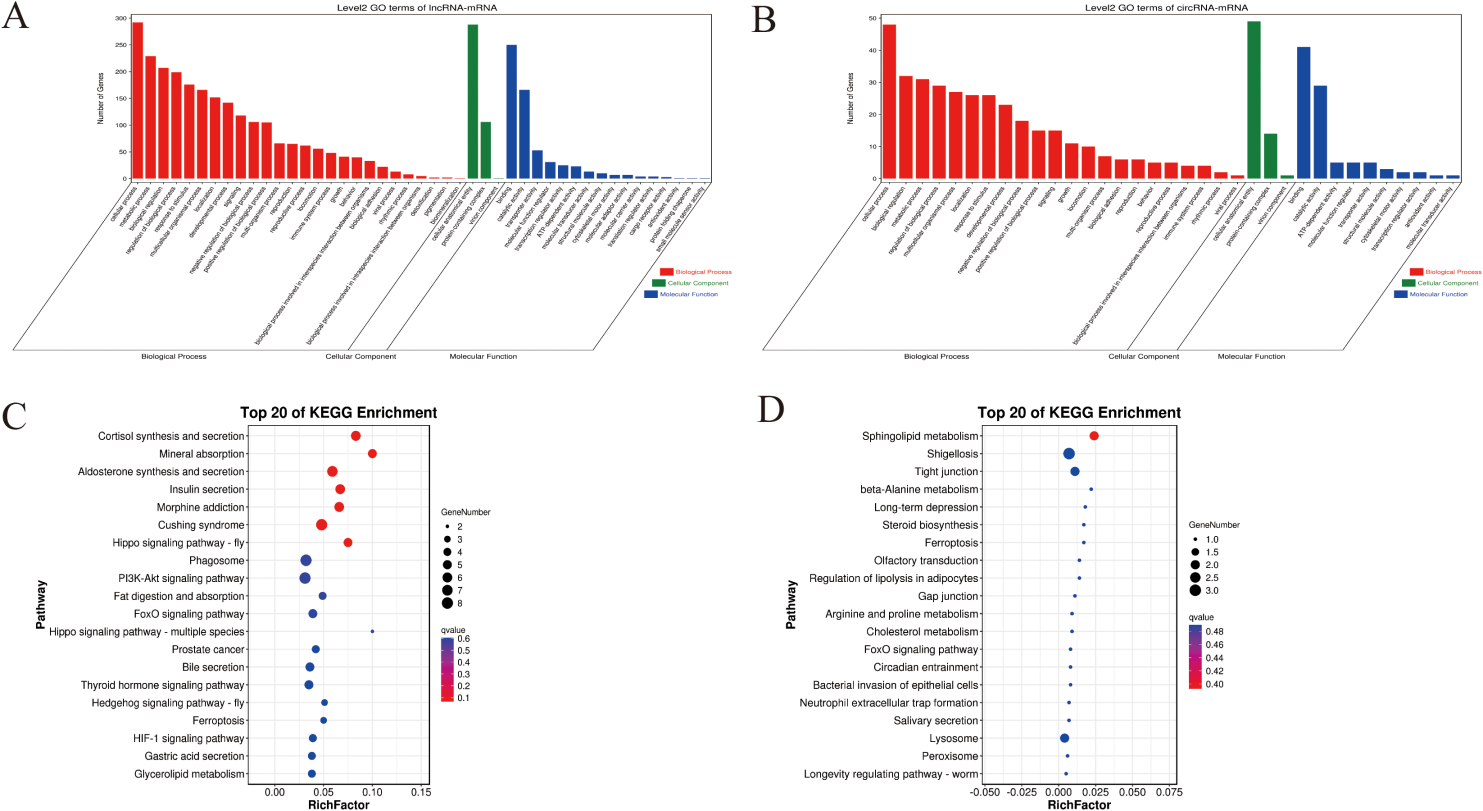
GO and KEGG enrichment for predicted target genes of the CeRNA-related lncRNAs and circRNAs in the different feeding stages of *R. haemaphysaloides.* **(A)** GO enrichment of target genes of the CeRNA-related lncRNAs. **(B)** GO enrichment of target genes of the CeRNA-related cirRNAs. **(C)** KEGG enrichment of the target genes of the CeRNA-related lncRNAs. **(D)** KEGG enrichment of the target genes of the CeRNA-related cirRNAs.

GO analysis showed that these differentially expressed mRNAs in the lncRNA-miRNA-mRNA and circRNA-miRNA-mRNA networks were related to cellular processes, metabolic processes, and biological regulation (Biological Process), Cellular Anatomical entity (cellular component), and protein binding (molecular function) (**Figures 6A, B**). KEGG pathway analysis identified 20 pathways enriched in these differentially expressed mRNAs involved in the lncRNA-miRNA-mRNA and circRNA-miRNA-mRNA networks. Notably, several pathways related to organ growth and development were identified: Insulin secretion, the Hippo signaling pathway, the Pl3K-Akt signaling pathway, the FoxO signaling pathway, and Ferroptosis in the lncRNA-miRNA-mRNA (**Figure 6C**), and Steroid biosynthesis, Cholesterol metabolism, the FoxO signaling pathway, and the Ferroptosis pathway in the circRNA-miRNA-mRNA network (**Figure 6D**). LncRNA MSTRG.12264.1, which exhibited strong connectivity, was enriched in the Pl3K-Akt signaling pathway and was related to apoptosis. CircRNA novel_circ_001885 in the FoxO signaling pathway was related to cholesterol metabolism.

### 3.5 CeRNA network analysis of pathways related to salivary glands

**Figure 7:**
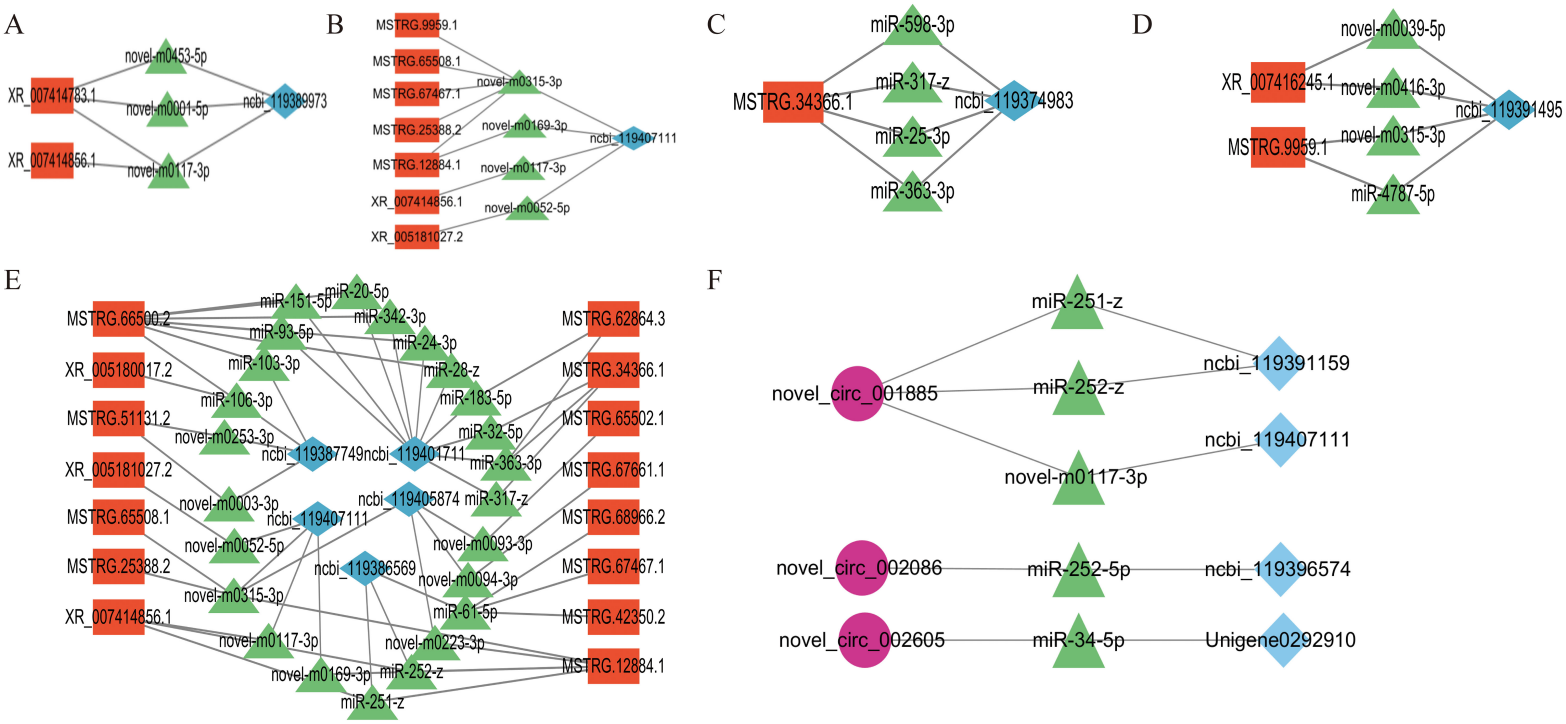
lncRNA–miRNA–mRNA regulatory network of signaling pathways related to salivary gland degeneration. **(A)** The insulin secretion signaling pathway **(B)** The FoxO signaling pathway. **(C)** The Hippo signaling pathway. **(D)** The Ferroptosis signaling pathway. **(E)** The Pl3K-Akt signaling pathway. **(F)** circRNA–miRNA–mRNA-related signaling pathways.

The results for cell growth and development-related pathways, the connectivity analysis identifying ceRNA relationships, and the KEGG enrichment results showed that Insulin secretion, the Hippo signaling pathway, the Pl3K-Akt signaling pathway, the FoxO signaling pathway, and the Ferroptosis pathway in the lncRNA-miRNA-mRNA (**Figures 7a–e**) involved the differential expression of the genes ncbi_119407111(Sgk3), ncbi_119401711(CrebA), ncbi_119386569(RAC1), ncbi_119387749(GNB5), ncbi_119405874(PKN2), ncbi_119389973(unc104), ncbi_119374983(Meis1), and ncbi_119391495(Gclm), as being involved in 14, 11, 8, 5,5, 4, 4, and 4 regulatory networks, respectively. The KEGG enrichment results identified Steroid biosynthesis, Cholesterol metabolism, the FoxO signaling pathway, and the Ferroptosis pathway in the circRNA-miRNA-mRNA network (**Figure 7f**), involving the differentially expressed genes ncbi_119391159(LIPF), ncbi_119401711(CrebA), ncbi_119396574 (shisa-5), and Unigene0292910 (ECR). In these differentially expressed genes, ncbi_119407111, ncbi_119401711, and ncbi_119386569 had strong connectivity in the lncRNA–miRNA–mRNA networks, and ncbi_119401711 was connected with lncRNA and circRNA, indicating their potential regulatory ability.

In brief, the network analysis demonstrated the multitarget effects of miRNAs, and the key regulatory factors in ceRNA networks were further identified.

### 3.6 Validation of lncRNA and mRNA expression

To validate the deep sequencing results, six miRNAs (miR-1-3p,miR-184-3p, miR-305-5p, novel-m0001-5p, novel-m0003-3p, and novel-m0004-3p) and six lncRNAs (XR_005181027.2, MSTRG.72349.3, MSTRG.34366.1, MSTRG.67467.1, MSTRG.12884.1, and MSTRG.65508.1) were randomly selected to detect their expression by qRT-PCR at the four stages.

**Figure 8:**
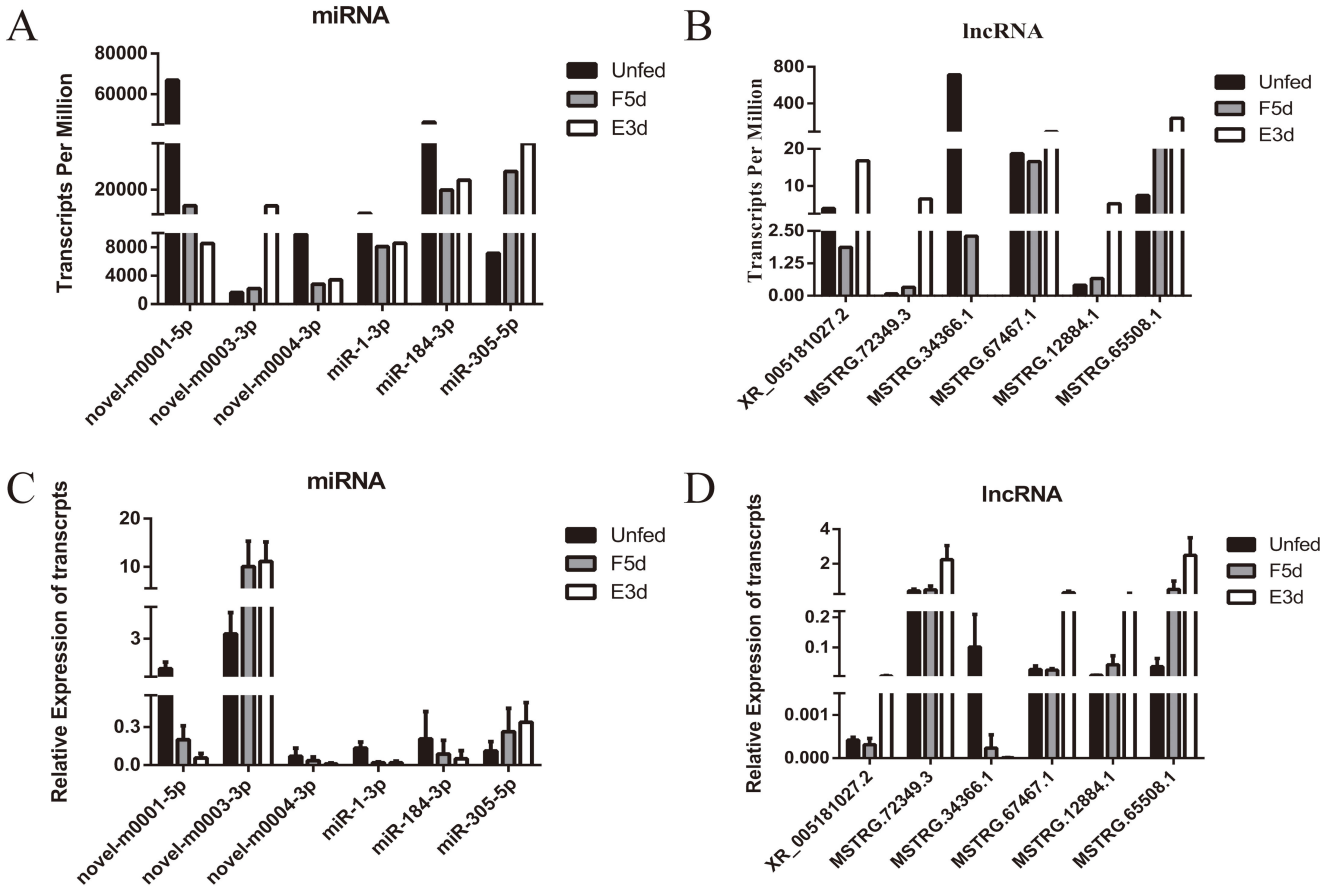
Validation of eight differentially expressed miRNAs and lncRNAs by qRT-PCR. **(A, B)** show the differentially expressed transcripts per million of miRNAs and lncRNAs from the sequencing data. **(C, D)** show the expression of selected miRNAs and lncRNAs as verified by qRT-PCR.

The qRT-PCR results of the six miRNAs and lncRNAs were consistent with the deep sequencing data (**Figures 8A–D**), thus confirming the findings.

## 4 Discussion

*R. haemaphysaloides* is a three-host hard tick widely distributed in China. This species is an important vector of several infectious pathogens (de la Fuente et al., 2008; Dantas-Torres et al., 2012; Ferreri et al., 2014). Tick-borne pathogens (TBPs) are transmitted to hosts through the saliva during feeding (Kazimírová and Stibrániová, 2013; Simo et al., 2017). The salivary glands of female ticks undergo degeneration after engorgement (Francischetti et al., 2009). Here, we report that mRNA and non-coding RNAs (lncRNA, miRNA, and circRNA) are related to salivary gland development in *R. haemaphysaloides*.

Several pivotal mRNAs and miRNAs have been reported to be involved in tick growth and development. During the degeneration of the salivary glands of *R. haemaphysaloides*, a recent study identified ATG5, Bcl-2, and three caspases (RhCaspases7, RhCaspases8, and RhCaspases9) involved apoptosis and autophagy pathways (Wang et al., 2020; Hu et al., 2021; Lu et al., 2021; Wang et al., 2021). Other studies have verified the involvement of novel-miRNA-hlo-miR-2 and let-7 related to molting events by targeting cuticular and ECR, and miR-184 and miR-275 related to blood digestion and oviposition by targeting vitellogenin (Hao et al., 2017; Malik et al., 2019; Wu et al., 2019; Liu et al., 2020b). However, neither lncRNAs nor circRNAs have been reported to be involved in tick growth and development.

During the slow feeding phase (days 1 to 5), the salivary glands of ticks develop rapidly, but they soon begin to degenerate during the rapid phase of feeding. In the present study, we simultaneously identified the lncRNA, circRNA, miRNA, and mRNA expression profiles in the unfed, F5d, and E3d groups using whole-transcriptome RNA-seq. We identified 449 lncRNAs, 73 circRNAs, and 499 miRNAs that were differentially expressed. Then, we constructed ceRNA regulatory networks that included 59 lncRNAs, 7 circRNAs, 110 miRNAs, and 394 mRNAs. In the network, there were 562 lncRNA-miRNA-mRNA and another 58 circRNA-miRNA-mRNA pairs.

Based on the transcriptome results, we constructed a ceRNA network to characterize the salivary gland development of the ticks. This ceRNA network revealed three pathways involved in the regulation of hormone production: insulin secretion, cholesterol metabolism, and steroid biosynthesis signal pathways. Four pathways, FoxO, Hippo, Ferroptosis, and Pl3K-Akt, are involved in the programmed cell death of the salivary glands. These findings align with the broader understanding of how organisms maintain homeostasis and adapt to environmental changes through precise regulation of growth, development, metabolism, and behavior. In *Drosophila*, the main factors regulating growth and development according to the environmental conditions in animals are the conserved insulin and insulin-like growth factors (IGFs) and steroid hormones (Hietakangas and Cohen, 2009; Tennessen and Thummel, 2011; Boulan et al., 2015). Insulin signaling, phosphatidylinositol 3-kinase (PI3K), and Akt (protein kinase B) mediate a series of signal events to promote cell survival or apoptosis (Clancy et al., 2001). In well-fed animals, phosphorylated Forkhead Box class O (FoxO) is excluded from the nucleus, thereby allowing growth to proceed, whereas under nutrient-restricted conditions, deactivation of Akt allows FoxO to enter the nucleus and act on its target genes, including 4E-binding protein, to suppress cell growth (Koyama et al., 2020). This process may be involved both before and after tick feeding, playing a significant role in unfed or well-fed stages. Moreover, insulin appears to govern ecdysone biosynthesis through effects on the Warts-Yorkie-bantam (Hippo) pathway that regulates the delivery of the steroid precursor cholesterol for ecdysone biosynthesis through an autophagosomal cholesterol-trafficking mechanism (Yan et al., 2016; Liu et al., 2020a). Specifically, ecdysone and its receptor EcR was demonstrated to regulate salivary gland degeneration by caspase-dependent apoptosis in the ecdysteroid signaling pathway of ticks (Mao and Kaufman, 1999b; Lu et al., 2021). Additionally, the Ferroptosis and Hippo signaling pathways play important roles in the feeding of ticks and the degeneration of the salivary glands (Galay et al., 2014; Xiao et al., 2020). Recent studies have identified key signaling pathways, including insulin signaling, the Hippo signaling pathway, and the Wnt signaling pathway, across various growth stages of the *Haemaphysalis longicornis* tick, underscoring their critical roles in regulating tick growth and development (Luo et al., 2019). These findings collectively highlight the complex interplay of signaling pathways in tick salivary gland development.

To facilitate future studies of the mechanisms involving lncRNAs and circRNA, we constructed a ceRNA network that included 59 lncRNAs targeting 104 miRNAs that in turn bound to 368 mRNAs, and 7 circRNA targeting 18 miRNAs that in turn bound to 58 mRNAs. MSTRG.66500.2, XR_007414783.1, XR_007414415.1, MSTRG.58344.1 XR_007414856.1, and novel_circ_001885 exhibited strong connectivity. The target genes of these lncRNAs and circRNA are involved in cell growth and development. Pde8b (ncbi_119388181, phosphodiesterase 8B) participates in the insulin metabolism pathway by regulating AMP (Dyachok et al., 2008; Rao et al., 2010); Creb (ncbi_119401711, cyclic AMP response element) regulates cell biological processes through the Pl3K-Akt signaling pathway (Lin et al., 2014); Sgk1 (ncbi_119407111, Serum and glucocorticoid regulated kinase 1) regulates cell autophagy through the FoxO signaling pathway (Yu et al., 2023). RhECR (Unigene0292910) and MEIS1(ncbi_119374983, homeobox protein homothorax) induce salivary gland cell apoptosis through caspase-dependent processes (Wermuth and Buchberg, 2005; Lu et al., 2021). GCLC(ncbi_119391495, glutamate-cysteine ligase) has a glutathione-independent, non-canonical role in the protection against ferroptosis by maintaining glutamate homeostasis under cystine starvation (Kang et al., 2021b). These molecules may play an important role in the development process of tick salivary glands.

Non-coding RNAs not only play a crucial role in regulating growth and development but are also integral to insect innate immunity. In response to *Metarhizium anisopliae* infection, multiple lncRNAs have been identified in *Plutella xylostella* that modulate the expression of immune-related mRNAs, including bGRP, toll, serpin, and transferrin (Guan et al., 2020). These lncRNAs may regulate genes involved in mosquito defense mechanisms, such as those encoding antimicrobial peptides and enzymes that target pathogens during microbial infections (Han et al., 2025). A ceRNA analysis has revealed a potential ceRNA regulatory network for *Drosophila* Toll immune responses, involving three lncRNAs and seven miRNAs (Huang et al., 2024). Given that ncRNAs, such as miRNAs and siRNAs, can be easily synthesized chemically and exhibit resilience against endogenous nuclease enzymes, the manipulation of ncRNA-mediated regulation of insect behavior holds promising potential for the development of effective pest management strategies. For instance, miRNAs have been successfully used to inhibit locust swarm formation through transgenic plants expressing short tandem target mimics (Yang et al., 2022). However, there are no reported studies on the roles of lncRNAs or circRNAs in tick immunity.

Notably, in our study of signaling pathways associated with the ceRNA network, such as insulin, FoxO, and PI3K-Akt pathways, evidence indicates their involvement in insect immune responses. For instance, *Bombyx mori* cell lines infected with *Bombyx mori* nuclear polyhedrosis virus exhibit increased Akt phosphorylation (Jin et al., 2018; Jiang et al., 2019) and reduced FOXO gene expression (Kang et al., 2021a). Additionally, feeding human insulin to *Anopheles stephensi* at concentrations comparable to those found in a blood meal activates the insulin signaling pathway and increases the prevalence of *Plasmodium falciparum* infection (Surachetpong et al., 2011; Pakpour et al., 2012; Pietri et al., 2016). Conversely, inhibiting insulin signaling pathway activity with a PI3K inhibitor reduces *P. falciparum* infection rates and upregulates immune effector gene expression in the mosquito midgut (Pakpour et al., 2012; Pietri et al., 2015).Based on these findings, we speculate that similar signaling pathways and related molecules may function analogously in ticks during infection with Babesia or other tick-borne viruses. Furthermore, these molecules may also be regulated by ceRNA mechanisms in ticks. Further research is needed to explore these potential regulatory networks and their implications for tick immunity.

In sum, this study provides complete data on the lncRNA and circRNA expression profiles of the salivary glands of *R. haemaphysaloides*. During salivary gland degeneration, many lncRNAs and circRNA undergo significant changes in expression levels. These lncRNAs and circRNAs, through the ceRNA network, influence signaling pathways that regulate the development of tick salivary glands. Furthermore, it is speculated that they may also play a role in modulating the immune function of ticks and their interactions with tick-borne pathogens. Finally, this study provides new perspectives concerning the involvement of CeRNA network in the degeneration of tick salivary glands through its effect on target genes. The findings suggest new ideas for the prevention and control of ticks and tick-borne diseases.

## Data Availability

The raw sequence data reported in this paper have been deposited in the Genome Sequence Archive (Genomics, Proteomics & Bioinformatics 2021) in National Genomics Data Center (Nucleic Acids Res 2024), China National Center for Bioinformation / Beijing Institute of Genomics, Chinese Academy of Sciences (GSA: CRA024860) that are publicly accessible at https://ngdc.cncb.ac.cn/gsa.
