# Supplementary material for: Transcriptome reveals the roles and potential mechanisms of CeRNA in the regulation of salivary gland development in the tick Rhipicephalus haemaphysaloides
Source: Front Cell Infect Microbiol. 2025 Apr 30;15:1573239. doi: 10.3389/fcimb.2025.1573239 (PMC12075121; doi:10.3389/fcimb.2025.1573239)
Supplement: Supplementary file 2 [file DataSheet2.pdf]

Supplementary Table S1

Statistics on data filtering and adapter Trimming of each sample for miRNA

id	clean_reads	high_quality	3'adapter_null	insert_null	5'adapter_contaminants	polyA	clean_tags
Unfed-1	14430941 (100%)	14290311 (99.0255%)	13953 (0.0976%)	135562 (0.9486%)	226026 (1.5817%)	1036 (0.0072%)	13594879 (94.2065%)
F5d-1	15635371 (100%)	15362950 (98.2577%)	330865 (2.1537%)	151561 (0.9865%)	71633 (0.4663%)	1128 (0.0073%)	14452234 (92.4329%)
E3d-1	9661137 (100%)	9553927 (98.8903%)	13740 (0.1438%)	100133 (1.0481%)	14212 (0.1488%)	371 (0.0039%)	8995262 (93.1077%)
Unfed-2	15063203 (100%)	14773096 (98.0741%)	342575 (2.3189%)	159342 (1.0786%)	9492 (0.0643%)	969 (0.0066%)	13876636 (92.1227%)
F5d-2	10061947 (100%)	9959614 (98.9830%)	67443 (0.6772%)	139011 (1.3957%)	21476 (0.2156%)	528 (0.0053%)	9521717 (94.6310%)
E3d-2	12751556 (100%)	12599030 (98.8039%)	11937 (0.0947%)	120465 (0.9561%)	20156 (0.1600%)	461 (0.0037%)	11747802 (92.1284%)
Unfed-3	11645650 (100%)	11474358 (98.5291%)	33408 (0.2912%)	149186 (1.3002%)	11467 (0.0999%)	619 (0.0054%)	10861207 (93.2641%)
F5d-3	12736954 (100%)	12570170 (98.6906%)	59497 (0.4733%)	276391 (2.1988%)	28776 (0.2289%)	1047 (0.0083%)	11617864 (91.2138%)
E3d-3	15387526 (100%)	15181492 (98.6610%)	48118 (0.3170%)	91753 (0.6044%)	17056 (0.1123%)	506 (0.0033%)	14458194 (93.9605%)

clean_reads : The small RNA data obtained after preliminary filtering of all raw data downloaded from the machine

high_quality : The remaining reads after filtering out low-quality reads (reads with a quality value below 20 and more than 1 alkali number or containing N) in the original data, with a ratio of high_quality/clean_reads;

3'adapter_null : The number of reads indicating that the 3-terminal connector was not found, with a ratio of 3'adapter/high_quality ；

insert_null : The number of reads indicating that the 3-terminal amd 5-terminal connector was not found, with a ratio of insert_null/high_quality ；

5'adapter_contaminants : The number of reads containing 5-terminal connectors, with a ratio of 5'adapter_contaminants/high_quality

polyA : The proportion of reads containing polyA in the small RNA tag obtained after removing the adapter is polyA/high_quality ；

clean_tags : The total amount of tags for the small RNA obtained after data processing, with a ratio of clean_tags/high_quality ；

Supplementary Table S2

Distribution of sRNAs in different categories for miRNA

sample	total	rRNA	snRNA	snoRNA	tRNA	known_mirna	novel_mirna	genome_others	unann
Unfed-1	10861207	339360	3736	757	19908	4810886	27661	1075694	4583205
Unfed-2	13594879	351127	1957	361	37448	6369774	714643	2029626	4089943
Unfed-3	13876636	380860	2085	438	30680	5926864	888149	2072926	4574634
**AVERAGE Unfed group**	**12777574**	**357115.7**	**2592.667**	**518.6667**	**29345.33**	**5702508**	**543484.3**	**1726082**	**4415927.333**
F5d-1	11617864	594402	8740	464	34639	4334523	59695	1763692	4821709
F5d-2	14452234	481260	10268	796	81116	6540203	259165	2771468	4307958
F5d-3	9521717	364222	9259	463	46037	3933410	155144	1802401	3210781
**AVERAGE F5d group**	**11863938**	**479961.3**	**9422.333**	**574.3333**	**53930.67**	**4936045.3**	**158001.3**	**2112520.33**	**4113482.667**
E3d-1	14458194	802268	8075	1521	79572	5444035	39168	2056582	6026973
E3d-2	8995262	440821	5164	1712	60569	4096858	189424	2029626	2870887
E3d-3	11747802	700135	6363	2389	73030	4052373	215993	1817095	4880424
**AVERAGE E3d group**	**11733753**	**647741.3**	**6534**	**1874**	**71057**	**4531088.7**	**148195**	**1967767.67**	**4592761.333**

Unfed: unfed：Not feeding; F5d :feed 5 days; E3d :engorged 3 days.

unann, rRNA, snoRNA, tRNA, snRNA, known miRNA and Novel mirna represent unannotate tags, ribosomal RNA, small nucleolar RNA, transfer RNA, small nuclear RNA, known microRNA and Novel microRNA respectively.

Supplementary Table S3
Base Information Statistics for lncRNA, circRNA and mRNA

**Sample**	Unfed-1	F5d-1	E3d-1	Unfed-2	F5d-2	E3d-2
**RawData(bp)**	11661815100	10481094900	10056693600	9816992700	9282423900	10001779800
**BF_Q20(%)**	10983945686 (94.19%)	9900043307 (94.46%)	9533320254 (94.80%)	9305254096 (94.79%)	8806416038 (94.87%)	9417896184 (94.16%)
**BF_Q30(%)**	10140442126 (86.95%)	9144343472 (87.25%)	8843567347 (87.94%)	8657449775 (88.19%)	8169479117 (88.01%)	8693200954 (86.92%)
**BF_N(%)**	720854 (0.01%)	690611 (0.01%)	652902 (0.01%)	208824 (0.00%)	829426 (0.01%)	869452 (0.01%)
**BF_GC(%)**	5787305266 (49.63%)	5282074874 (50.40%)	4988457125 (49.60%)	4912545466 (50.04%)	4623073412 (49.80%)	4873378909 (48.73%)
**CleanData(bp)**	11492595412	10335238385	9903462489	9647317046	9131326300	9822722167
**AF_Q20(%)**	10859626952 (94.49%)	9791670891 (94.74%)	9416223832 (95.08%)	9181528045 (95.17%)	8691027835 (95.18%)	9283909516 (94.51%)
**AF_Q30(%)**	10035189725 (87.32%)	9051909518 (87.58%)	8743123973 (88.28%)	8551684502 (88.64%)	8070131052 (88.38%)	8579086499 (87.34%)
**AF_N(%)**	239532 (0.00%)	221285 (0.00%)	213160 (0.00%)	151493 (0.00%)	259300 (0.00%)	276868 (0.00%)
**AF_GC(%)**	5691656808 (49.52%)	5199055932 (50.30%)	4901724441 (49.50%)	4812861051 (49.89%)	4541292220 (49.73%)	4777744802 (48.64%)

BF: Before filter, Sample base information before filtering.
AF: After filter, Sample base information after filtering.
Raw Data(bp): Total number of base pairs in offline data (unit: bp).
Clean Data(bp): Total number of bases in high-quality filtered data (unit: bp).
Q20(%): The number of bases with sequencing base quality values above Q20 and their percentage in RawData (or CleanData).
Q30(%): The number of bases with a quality value of Q30 or above in sequencing and their percentage in RawData (or CleanData).
N (%): The number of N bases in single-end reads and their percentage in RawData (or CleanData).
GC (%): GC ratio of sequence bases before (after) filtering.

Supplementary Table S4

Comparison of reference statistics for lncRNA, circRNA and mRNA

Sample	Total	Unmapped(%)	Unique_Mapped(%)	Multiple_Mapped(%)	Total_Mapped(%)
Unfed-1	74928428	30483807 (40.68%)	43210125 (57.67%)	1234496 (1.65%)	44444621 (59.32%)
F5d-1	67423968	22077569 (32.74%)	44233712 (65.61%)	1112687 (1.65%)	45346399 (67.26%)
E3d-1	63850682	22739949 (35.61%)	40072031 (62.76%)	1038702 (1.63%)	41110733 (64.39%)
Unfed-2	62661338	25163455 (40.16%)	36140892 (57.68%)	1356991 (2.17%)	37497883 (59.84%)
F5d-2	60053770	18672245 (31.09%)	40341384 (67.18%)	1040141 (1.73%)	41381525 (68.91%)
E3d-2	64512770	24827336 (38.48%)	38711077 (60.01%)	974357 (1.51%)	39685434 (61.52%)

Unmapped(%):The number of reads not compared to the reference genome and the proportion of valid reads
Unique_Mapped(%):The number of reads that are uniquely compared to the reference genome and the proportion of valid reads
Multiple_Mapped(%):Comparing the number of reads and their proportion to the effective reads of the reference genome across multiple locations
All_Mapped(%):The total number of reads that can be mapped to the genome and their proportion relative to the effective reads.

Supplementary Table S5. The primers of lncRNA used in this study

Name of primers	Sequences (from 5′ to 3′)
XR_005181027.2-F	CCATCACCTGGAAACTGAGC
XR_005181027.2-R	AGATATGGATGGCTGCTGCT
MSTRG.72349.3-F	CCGTTTCGCTAGCTTGACAT
MSTRG.72349.3-R	ATGAGCGTGGCGTGTAAATC
MSTRG.34366.1-F	AGCCTTCACGGATTGGTTGC
MSTRG.34366.1-R	CATGTGGCATTCGCCAGGTA
MSTRG.67467.1-F	ATGCCAACCAACTTGCCGAA
MSTRG.67467.1-R	AGGTTACCTTCGTGCATGGC
MSTRG.12884.1-F	CACTACTTACGGCGACAACC
MSTRG.12884.1-R	GGAAATCGCAGTCCATCGAG
MSTRG.65508.1-F	CTGAAACATGCGGAGACCAG
MSTRG.65508.1-R	CGGGACAAGGAGATACACGA
ELF1A-F	CGTCTACAAGATTGGTGGCATT
ELF1A-R	CTCAGTGGTCAGGT TGGCAG

Abbreviations: F, forward primer; R, reverse primer.

Table S6. The primers of miRNAs used in this study

Name of primers	Sequences (from 5′ to 3′)
novel-m0001-5p	CTTGGGCTGAAGTTCGTCGTTG
novel-m0003-3p	TCCGGCGTGGTCTAGTGGC
novel-m0004-3p	ACCTGCACACGACTGCTTGACT
miR-184-y	TGGACGGAGAACTGATAAGG
miR-305-x	ATTGTACTTCATCAGGTGCTCTGG
miR-1-y	TGGAATGTAAAGAAGTATGGAG

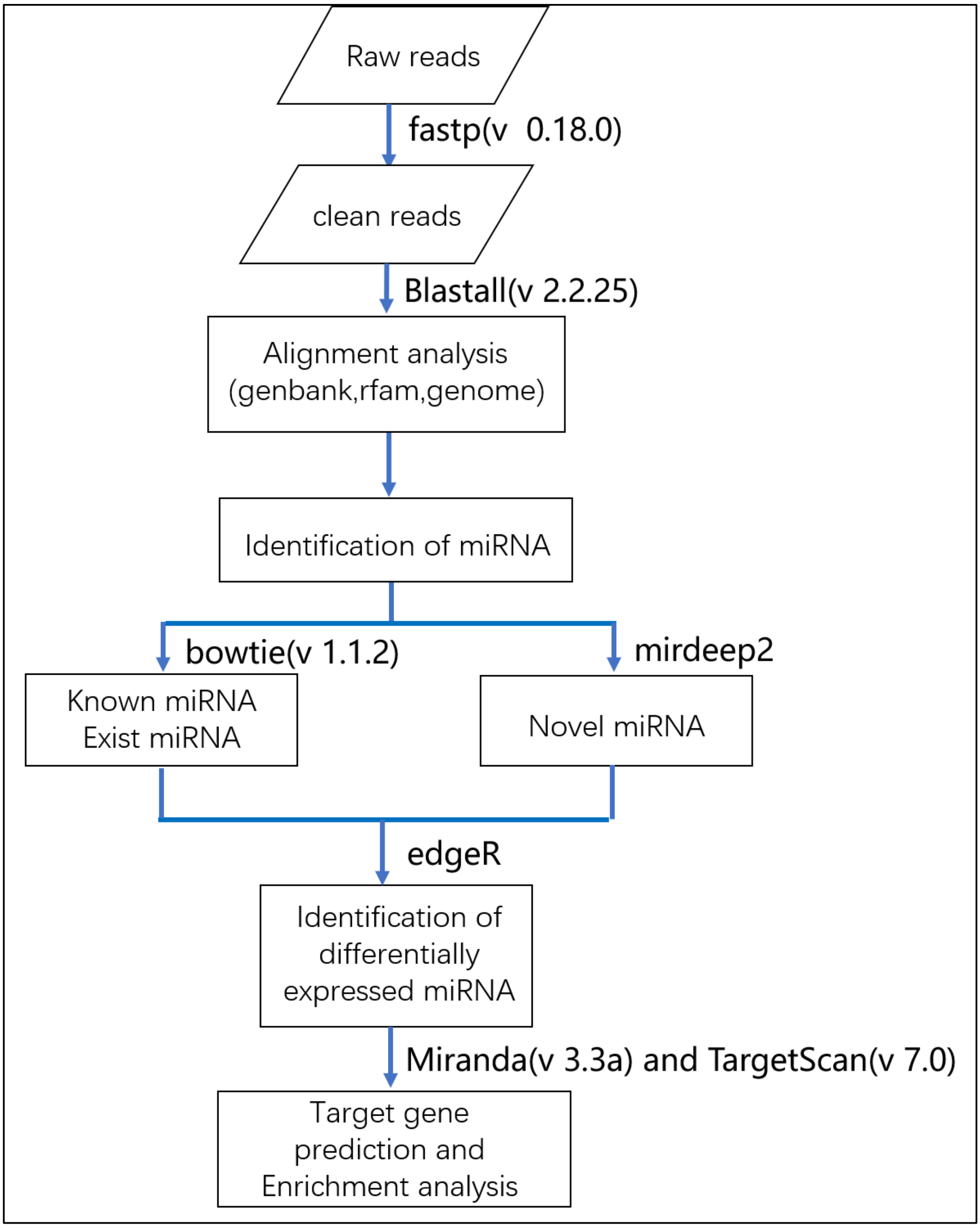

Supplementary Fig.S1: Workflow diagram of data analysis for miRNA

Filtering of Clean Tags Reads obtained from the sequencing machines included dirty reads containing adapters or low quality bases which would affect the following assembly and analysis by fastp (v0.18.0). Thus, to get clean tags, raw reads were further filtered according to the following rules: (1) Removing low quality reads containing more than one low quality (Q-value≤20) base or containing unknown nucleotides(N); (2) Removing reads without 3’ adapters; (3) Removing reads containing 5’ adapters; (4) Removing reads containing 3’ and 5’ adapters but no small RNA fragment between them; (5)

Removing reads containing ploy A in small RNA fragment; (6) Removing reads shorter than 18nt (not include adapters)
Alignment analysis (genebank, rfam, genome) by Blastall (v2.2.25): (1) All of the clean tags were aligned with small RNAs in GeneBank database (Release 209.0) to identify and remove rRNA,scRNA,snoRNA,snRNA and tRNA. (2) Meanwhile all of the clean tags were aligned with small RNAs in Rfam database (Release 11.0) to identify and remove rRNA,scRNA,sonRNA,snRNA and tRNA. (3) All of the clean tags were also aligned with reference genome. Those mapped to exons or introns might be fragments from mRNA degradation, so these tags were removed. The tags mapped to repeat sequences were also removed.
Identification of miRNA: All of the clean tags were then searched against miRBase database (Release 22) to identify known (Species studied) miRNAs (exist miRNAs) by bowtie (v 1.1.2). So far the miRNA sequences of some species were still not included in miRBase database. For those species the miRNAs alignment with other species was a dependable way to identify the known miRNAs. All of the unannotated tags were aligned with reference genome. According to their genome positions and hairpin structures predicted by software mirdeep2, the novel miRNA candidates were identified.
DE miRNA Analysis: miRNAs differential expression analysis was performed by edgeR software between two different groups or samples.We identified miRNAs with a fold change ≥2 and P value <0.05 in a comparison as significant DE miRNAs.

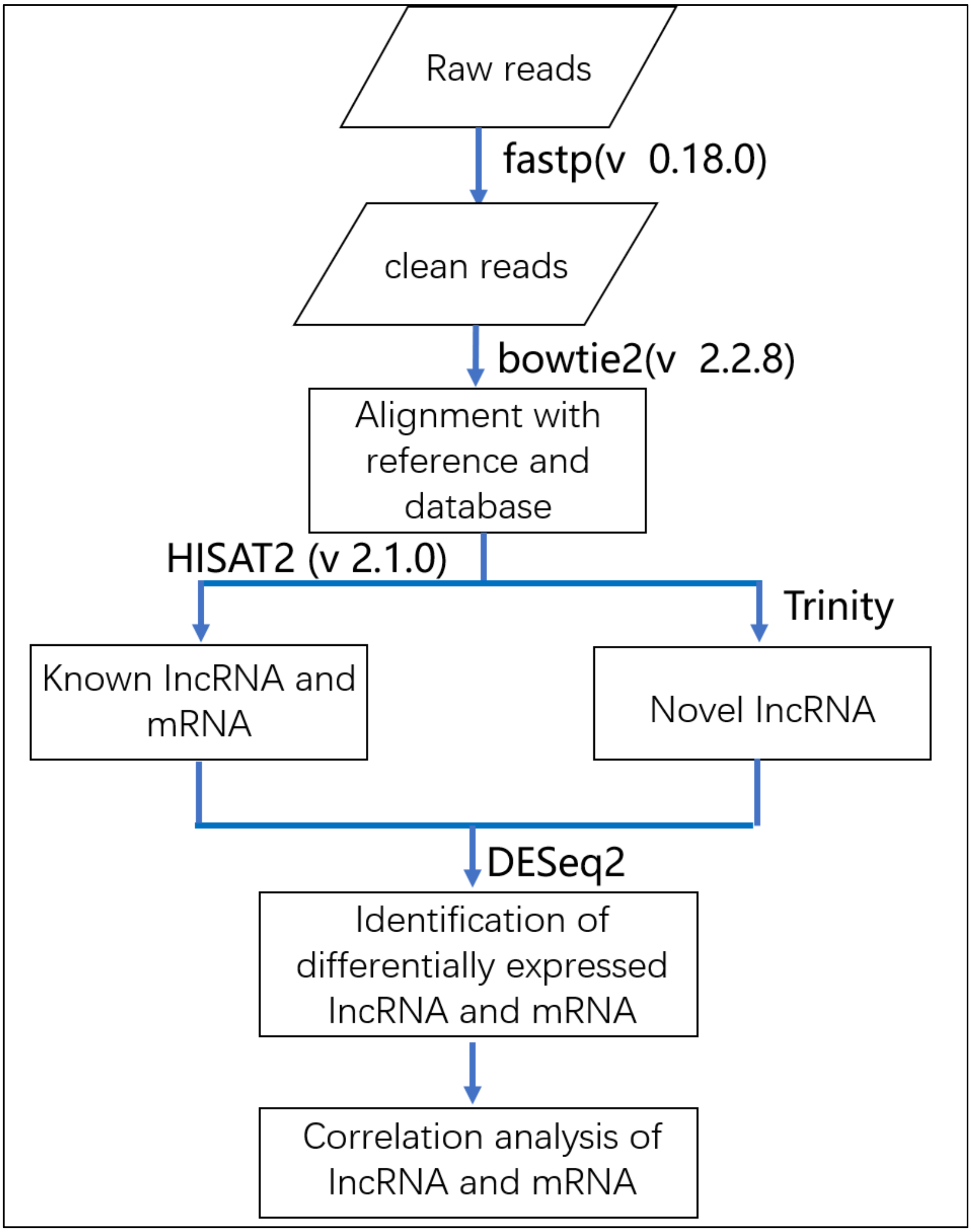

Supplementary Fig.S2: Workflow diagram of data analysis for lncRNA, circRNA and mRNA.

Filtering of Clean Reads: Reads obtained from the sequencing machines included raw reads containing adapters or low quality bases which would affect the following assembly and analysis. Thus, to get high quality clean reads, reads were further filtered by fastp (version 0.18.0). The parameters were as follows: (1)removing reads containing adapters; (2)removing reads containing more than 10% of unknown nucleotides (N);(3)removing low quality reads

containing more than 50% of low quality (Q-value≤20) bases.

Alignment with reference and database: Short reads alignment tool Bowtie2 (version 2.2.8) was used for mapping reads to ribosome RNA (rRNA) database. The rRNA mapped reads were then removed. The remaining reads were further used in assembly and analysis of transcriptome. An index of the reference genome was built, and paired-end clean reads were mapped to the reference genome using (version 2.1.0) with "-rna-strandness RF" and other parameters set as a default.

Alignment with reference genome: An index of the reference genome was built, and paired-end clean reads were mapped to the reference genome using HISAT2 (version 2.1.0) with "-rna-strandness RF" and other parameters set as a default. The reconstruction of transcripts was carried out with software Stringtie (version 1.3.4), which together with HISAT2 and Trinity, allow biologists to identify new genes and new splice variants of known ones.

Differentially expressed transcripts (DEGs) Analysis: The differentially expressed transcripts of coding RNAs and lncRNAs/circRNA were analyzed respectively. RNAs and lncRNAs differential expression analysis was performed by DESeq2 software between two different groups. The genes/transcripts with the parameter of false discovery rate (FDR) below 0.05 and absolute fold change≥2 were considered differentially expressed genes/transcripts. Differentially expressed coding RNAs were then subjected to enrichment analysis of GO functions and KEGG pathways.
